# Effects of sodium chloride and sodium tripolyphosphate on the prooxidant properties of hemoglobin in washed turkey muscle system

**DOI:** 10.1016/j.fochx.2022.100480

**Published:** 2022-10-13

**Authors:** Haizhou Wu, Sung Yong Park, Mark P. Richards

**Affiliations:** a Department of Animal and Dairy Sciences, University of Wisconsin-Madison, Meat Science and Animal Biologics Discovery, 1933 Observatory Dr. Madison, WI 53706, United States; b National Center of Meat Quality, Safety Control, Jiangsu Innovation Center of Meat Production, Processing, College of Food Science, Technology, Nanjing Agricultural University, Nanjing 210095, PR China; c Department of Biology and Biological Engineering–Food and Nutrition Science, Chalmers University of Technology, SE 412 96 Gothenburg, Sweden

**Keywords:** Heme protein, Meat, NaCl, STPP, Hemin, Auto-oxidation, pH

## Abstract

This study examined the effects of sodium chloride (NaCl) and sodium tripolyphosphate (STPP) on lipid oxidation induced by oxyhemoglobin (oxyHb) in washed turkey muscle (WTM) model. To explore the reasons for observed effects, the pro-oxidant abilities of Hb derivatives (e.g., metHb, oxyHb, hemin, Fe^2+^, and Fe^3+^), pH change, and antioxidation of Hb in the presence of NaCl or STPP were also analyzed. The observed lipid oxidation capacity in WTM followed the order metHb > hemin > oxyHb > Fe^2+^ > Fe^3+^. Added Fe^2+^ accelerated auto-oxidation of oxyHb and oxyHb-mediated lipid oxidation. Hb auto-oxidation to metHb increased as the pH decreased from 6.6 to 5.0. NaCl promoted oxyHb-mediated lipid oxidation due to NaCl causing decreased pH value and increased formation of metHb. STPP inhibited oxyHb-mediated lipid oxidation and weakened the pro-oxidative effect of NaCl. This could be attributed to STPP increasing the pH, inactivating free iron, and inhibiting formation of metHb.

## Highlights

• MetHb in WTM acted as the most effective pro-oxidant, followed by hemin and oxyHb.
• The addition of NaCl significantly increased the oxyHb-mediated lipid oxidation.
• STPP inhibited oxyHb-mediated lipid oxidation.
• Formation of metHb and pH paly critical roles in oxyHb-mediated lipid oxidation.

## 1 Introduction

Lipid oxidation is a major cause of quality deterioration affecting flavor, color, texture, nutritional value, and safety of meat and meat products (Wu, Richards, & Undeland, 2022). This process is affected by several ante- and post-mortem factors, including the level and state (ferric or ferrous) of muscle pro-oxidants (hemoglobin, myoglobin, free ionic iron) and antioxidant levels (α-tocopherol) (Wu, Forghani, Abdollahi, & Undeland, 2022). Likewise, the activity and level of antioxidant enzymes (glutathione peroxidase, superoxide dismutase, and catalase) and the composition of muscle fat are added important factors affecting lipid oxidation (Richards, 2010, Wu et al., 2022). Furthermore, processing procedures (adding sodium chloride, sodium tripolyphosphate, and cooking) and storage conditions (packaging method and storage temperature) are other environmental factors affecting lipid oxidation in meat and meat products (Domínguez, Pateiro, Gagaoua, Barba, Zhang, & Lorenzo, 2019).

Various non-meat ingredients are used to manufacture processed meats to enhance their flavor and texture. Sodium chloride (NaCl), or salt, is added to meat products to improve flavor, preservation, water holding capacity, tenderness, and juiciness (Wu et al., 2015). Nevertheless, it can act as a pro-oxidant in meat and meat products (Mariutti & Bragagnolo, 2017). However, the pro-oxidant effects of NaCl depend on its concentration levels. A low concentration of NaCl increases lipid oxidation, while a high concentration inhibits this process (Erickson, 2002). Possible theories related to the lipid oxidation mechanism with NaCl are that it promotes iron ion release from heme proteins (e.g., hemoglobin and myoglobin) in muscle (Rhee & Ziprin, 2001), which is a process known to initiate lipid auto-oxidation, and that it inhibits activities of antioxidant enzymes (Hernández, Park, & Rhee, 2002).

Sodium tripolyphosphate (STPP) is commonly added to meat products and is used for its similar effects to NaCl in textural properties of meat products (Thangavelu, Kerry, Tiwari, & McDonnell, 2019). However, STPP has been reported to have antioxidative properties in meat and meat products, differing from the known activity of NaCl (Maestre et al., 2009, Yuan et al., 2022). Researchers believe that STPP inhibits lipid oxidation in muscle food due to increased tissue pH and chelating free metal ions. For example, in raw beef rolls, adding STPP (0.5 %) increases the pH values from 5.32 to 5.80 and reduces the formation of metmyoglobin and thiobarbituric acid reactive substances (TBARS), resulting in about 15 and 78 % reductions, respectively (Lee, Hendricks, & Cornforth, 1998). Additionally, Maestre et al. (2009) studied the effects of chelators [ethylenediaminetetraacetic acid (EDTA), citric acid, STPP, and adenosine-5-triphosphate (ATP)] in the prevention of lipid oxidation mediated by fish hemoglobin. These findings identified STPP (2 mM) as the iron chelator with a significant efficiency in inhibiting lipid oxidation mediated by Pollock hemoglobin.

Although recent developments have been significant, it is challenging to elucidate how NaCl accelerates lipid oxidation while STPP inhibits this process activated by heme proteins in meat and meat products. These challenges exist due to simultaneous and continuous complicated phenomena such as heme protein auto-oxidation, heme dissociation, ferryl radical formation, and iron release by heme destruction. Therefore, this study aimed to estimate the effects of NaCl and STPP on lipid oxidation mediated by hemoglobin using washed turkey muscle (WTM) and explore the reasons for those effects.

## 2 Materials and methods

### 2.1 Chemicals

Ferric chloride, streptomycin sulfate, ferrous sulfate, barium chloride, ammonium thiocyanate, and sucrose were obtained from Sigma-Aldrich (St. Louis, MO). Chloroform (ethanol stabilized), methanol, and tris-(hydroxymethyl)-aminomethane (Tris) were obtained from Fisher Scientific (Pittsburgh, PA). All other chemicals used were analytical grade.

### 2.2 Preparation of hemolysate

Pig blood was obtained under Wisconsin State Inspection in the UW-Madison Meat Science and Muscle Biology Laboratory. Four volumes of pig blood were mixed thoroughly with 1 vol of anticoagulant containing 150 mM NaCl and sodium heparin (120 Units/ml). To remove the plasma, washing buffer (4 volumes of 1.7 % NaCl in 1 mM Tris, pH 8.0) were added to heparinized blood and centrifuged (700 *g* for 10 min at 4 °C) in a Beckman J-6B centrifuge (Beckman Instruments Inc., Palo Alto, CA). The red blood cells were washed three times more using 10 volumes of the same washing buffer (Fyhn, Fyhn, Davis, Powers, Fink, & Garlick, 1979). After which, 3 volumes of stock solution (1 mM Tris, pH 8.0) were added to lyse cells for 30 min. One-tenth volume of 1 M NaCl was then added to aid in stromal removal, and it was centrifuged (28,000 *g* for 15 min at 4 °C) in a Beckman L8-70 M ultracentrifuge (Beckman Instruments Inc., Palo Alto, CA). Hemolysates were then stored at −80 °C until use.

### 2.3 Quantification of hemoglobin level

To measure hemoglobin concentration, hemolysates were diluted in 1 mM Tris, pH 8.0. The diluted hemolysates were scanned from 700 to 400 nm in a model UV-2401 dual-beam spectrophotometer (Shimadzu Scientific Instruments Inc., Columbia, MD) using 1 mM Tris, pH 8.0 as a reference. Hemoglobin concentrations were based on a heme basis and calculated using the peak absorbance occurring near 578 nm (Zijlstra & Buursma, 1997).

### 2.4 Preparation of methemoglobin (metHb)

Hemoglobin (Hb) was converted into metHb by adding potassium ferricyanide and incubating on ice for 10 min (Wu, Yin, Zhang, & Richards, 2017). Ferricyanide was removed using DG-10 desalting columns (Bio-Rad, Hercules, CA) (Wu, Yin, Xiao, Zhang, & Richards, 2022), and isolated MetHb was stored at −80 °C until use.

### 2.5 Preparations of WTM

Turkey (*Meleagris gallopavo*) muscle was obtained from Kraft Oscar Mayer (Newberry, SC). WTM was prepared according to the method described by Wu et al. (2021b). Specifically, turkey muscles were trimmed and cut into small pieces to remove all bones, dark tissue, and remaining blood. The pieces were ground using a Kitchen Aid, Inc. (St. Joseph, MI) KSM90WW household mixer equipped with a grinding apparatus (5 mm plate diameter). Ground turkey muscles were washed 6 times using 50 mM sodium citrate buffer (pH 5.6), and at each washing time, turkey muscles were mixed with citrate buffer for 2 min with a glass rod, soaked for 15 min, and then dewatered using a fiberglass screen. After the final soak, muscle slurry was homogenized for approximately 3 min with a Polytron Type PT 10/35 probe (Brinkmann Instruments, Westbury, NY), then centrifuged at 15,263 g for 25 min (SORVALL RC5C PLUS). Collected pellets were stored at −80 °C in vacuum-sealed plastic bags. All washing, dewatering, and centrifugation steps were performed at 4 °C.

### 2.6 Addition of Hb derivatives, NaCl, and STPP to turkey muscle

WTMs were moved into an amber bottle (30 mL capacity). The Hb derivatives (oxyHb, metHb, hemin, ferrous iron, or ferric iron; 40 μmol Fe per kg WTM), NaCl (1.5 %), and STPP (0.3 %) were added to the mixture and mixed for 2 min using a plastic spatula. Following this mixture, streptomycin sulfate (200 ppm) was added to inhibit microbial growth (Wu, Xiao, Yin, Zhang, & Richards, 2021a). The moisture content was then adjusted to 90 % in the final system. The pH of WTM systems was measured, and samples were stored at 4 °C during storage.

### 2.7 Measuring auto-oxidation rate

The rate of metHb formation was calculated from absorbance changes at 540, 560, and 576 nm as described previously (Benesch, Benesch, & Yung, 1973). To confirm the effects of pH on metHb formation, oxy-form Hb (40 uM) was used and adjusted to a pH of 5.0, 5.4, 5.8, 6.2, and 6.6 using 100 mM bis-tris buffer adjusted to each pH. Three NaCl levels (1, 1.5, and 2.0 %), three STPP levels (0.1, 0.3, and 0.5 %) and one combination (NaCl 1.5 % and STPP 0.3 %) were added to oxyHb buffered with 100 mM bis-tris (pH 5.8). Three mM of superoxide dismutase and catalase per M of heme were added to the mixture to remove any superoxide and hydrogen peroxide formation.

### 2.8 Measuring hexanal level

Hexanal from WTM was extracted using a solid-phase microextraction (SPME) technique. Samples (1 g) were transferred into a 10 mL vial with 20 mm clear crimp. The vial was sealed with a metal hole cap with PTFE/silicone septa (MicroLiter Analytical Supplies Inc., Suwanee, USA). Before extraction of hexanal, fiber (65 um, Polydimethylsiloxane -divinylbenzene, Supelco, Bellefonte, USA) was conditioned by heating in a gas chromatograph (GC, HP 6890, Hewlett-Packard, Palo Alto, USA) injection port equipped with capillary column (DB-5, 30 m length × 0.25 mm i.d. × 0.1 uL film thickness) and flame ionization detector (FID) at 260 °C for 30 min. Vials containing samples were preheated for equilibration at 40 °C for 5 min, and SPME fiber for extraction of hexanal was exposed to the headspace above the sample at 40 °C for 10 min. After injection of SPME fiber into the GC/FID injection port, hexanal extracted from samples were isolated from SPME fiber at 250 °C for 5 min. Helium as a carrier gas and splitless mode was used. The flow rate of the carrier gas was 1 mL/min. Inlet and detector temperatures were 250 and 270 °C, respectively. The oven temperature was programmed at 40 °C for 5 min with a 10 °C/min ramp rate until 90 °C. Hexanal was identified by comparison of retention time of hexanal standard (Sigma-Aldrich, Steinheim, Germany) in GC/FID. The quantity of hexanal in the sample was calculated using an external standard method. Hexanal solutions prepared in different concentrations in Milliq water were used to make the standard curve. The hexanal solutions (1 mL) were analyzed using the same procedures described for the samples above.

### 2.9 Determination of TBARS

Thiobarbituric acid reactive substance (TBARS) values were measured according to the method described by Lemon (1975). An approximately 1 g sample was mixed with 5 mL of trichloroacetic acid reagent (7.5 % trichloroacetic acid, 0.1 % disodium ethylenediaminetetraacetic acid, and 0.1 % propyl gallate) and homogenized with a Polytron Type (PTA) 20/2 W probe (Brinkmann Instruments, Westbury, NY) for 30 *sec*. The homogenate was then filtered with Whatman no. 1. About 1 mL of filtrate and 1 mL of thiobarbituric acid solution (0.02 M) were mixed and incubated at 100 °C for 40 min. After cooling, the mixture was centrifuged at 2,000 *g* for 5 min at 4 °C using a Beckman J-6B centrifuge (Beckman Instruments Inc., Palo Alto, CA). Absorbance was then measured at 532 nm.

### 2.10 Statistical analysis

Data were analyzed using a one-way analysis of variance (ANOVA) with SPSS 12.0. The results were reported as mean ± standard deviation (SD) (n ≥ 2). Means were separated using Student-Newman-Keuls's multiple range test. The threshold for significance for all tests was set at *p* < 0.05.

## 3 Results

### 3.1 The pH value of WTM in the presence of NaCl and STPP

Table 1 shows the effects of NaCl (1.5 %) and STPP (0.3 %) on the pH values of WTM with or without oxyHb, metHb, hemin, ferrous and ferric iron. In general, the addition of oxyHb, metHb, hemin, ferrous and ferric iron did not significantly influence the pH values of WTM. However, the addition of both NaCl and STPP showed significant effects on the pH values as follows STPP > NaCl + STPP > Control > NaCl (Table 1).

**Table 1:** Effects of NaCl (1.5%) and STPP (0.3%) on pH values of washed turkey muscle (WTM) with added oxyHb, metHb, hemin, ferrous and ferric iron.

Treatments	Control	NaCl	STPP	NaCl + STPP
WTM	5.93 ± 0.00^C^	5.73 ± 0.00^D^	6.33 ± 0.01^A^	6.12 ± 0.00^B^
WTM + oxyHb	5.95 ± 0.01^C^	5.74 ± 0.01^D^	6.33 ± 0.01^A^	6.11 ± 0.01^B^
WTM + metHb	5.95 ± 0.01^C^	5.74 ± 0.01^D^	6.33 ± 0.01^A^	6.11 ± 0.01^B^
WTM + hemin	5.95 ± 0.00^C^	5.71 ± 0.01^D^	6.36 ± 0.01^A^	6.14 ± 0.01^B^
WTM + ferrous iron	5.95 ± 0.01^C^	5.71 ± 0.00^D^	6.31 ± 0.03^A^	6.09 ± 0.01^B^
WTM + ferric iron	5.94 ± 0.00^C^	5.72 ± 0.01^D^	6.33 ± 0.01^A^	6.11 ± 0.01^B^

Each oxidant provided 40 µmol Fe per kg WTM (10 µmol Hb/kg WTM, 40 µmol Mb/kg WTM, 40 µmol hemin/kg WTM, 40 µmol ferrous chloride/kg WTM, and 40 µmol ferric chloride/kg WTM).

### 3.2 Auto-oxidation of Hb due to pH, NaCl, STPP, and Fe^2+^

**Fig. 1:**
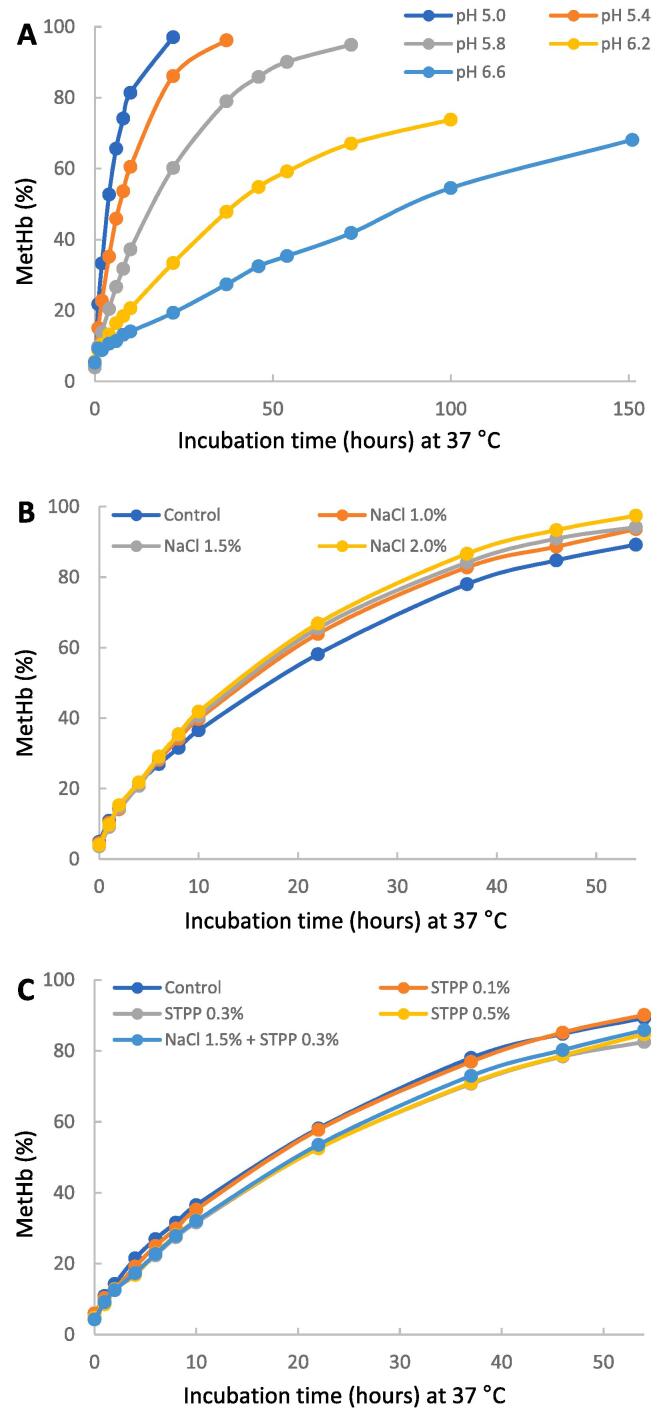
Relative rates of auto-oxidation (metHb %) of Hb during incubation at 37 °C, as affected by pH (A), NaCl (B) and STTP (C). The Hb concentration was 10 μM. Solutions were buffered in 100 mM bis-tris (pH 5.0 to 6.6) and contained 3 mmol superoxide dismutase and catalase per mol of heme. The pH was 5.8 for Hb solutions containing added NaCl and STPP.

Effects of pH, NaCl, and STPP on the auto-oxidation rate of oxyHb were measured while stored at 37 °C, and data are expressed as relative rates of auto-oxidation (metHb %). As shown in Fig. 1A and Supplementary material Table S1, auto-oxidation rates of oxyHb were pH-dependent: Hb solution with a high pH had a lower (p < 0.05) metHb concentration than solutions with low pH. At pH 5.8, Hb solutions with NaCl (1.0 ∼ 2.0 %) had greater rates of metHb formation than control (Fig. 1B and Supplementary material Table S2), while 0.3 % STPP reduced metHb formation from oxyHb (Fig. 1C and Supplementary material Table S2). In addition, STPP at 0.3 % masked the negative effects of 1.5 % NaCl on metHb formation (T7 in Supplementary material Table S2).

**Fig. 2:**
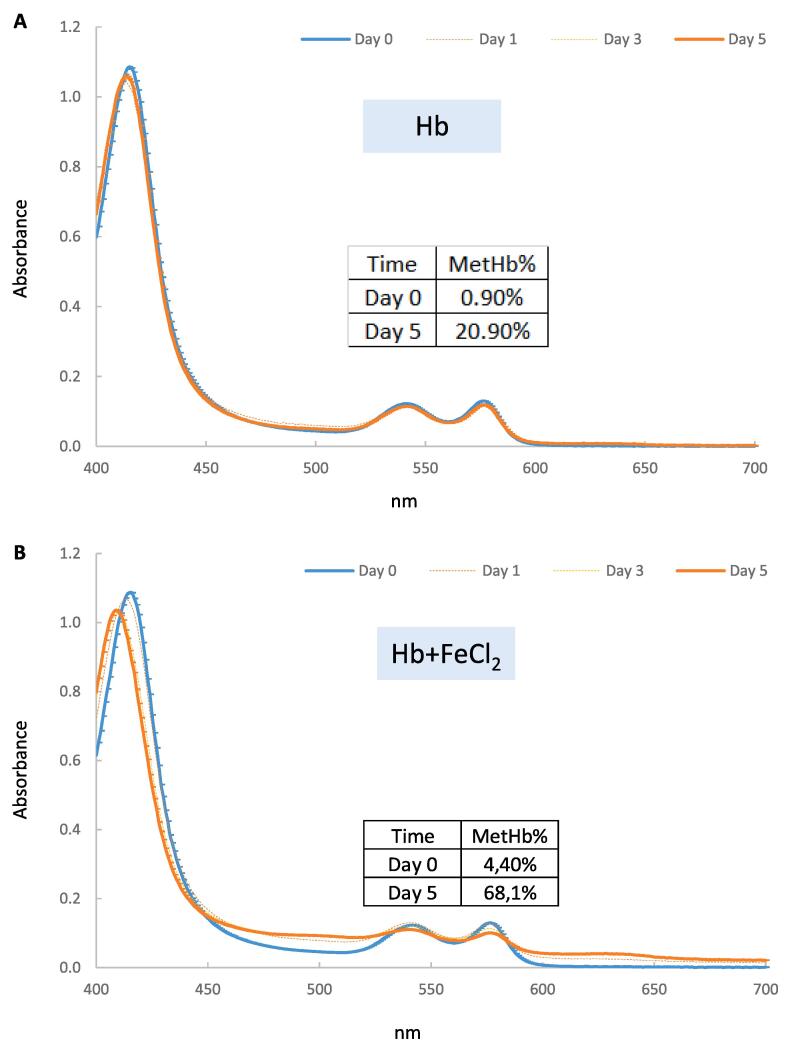
Time courses for UV–visible spectra change of bovine oxyHb (A) or bovine oxyHb + FeCl_2_ (B) during storage at 4 °C at pH 5.9. The concentrations of Hb and FeCl_2_ were 10 µM and 40 µM, respectively. Samples were diluted 5 times before scanning by 100 mM bis-tris (pH 5.9).

The ability of added Fe 2 + to facilitate Hb auto-oxidation was also examined at 4 °C at pH 5.9 (Fig. 2). The oxyHb with Fe^2+^ showed a large shift from 415 nm to 405 nm. Whereas in the oxyHb only treatment, only very slight shift at 415 nm was observed during 5 days incubation. The metHb was 68.1 % in oxyHb with FeCl_2_ and 20.9 % in only oxyHb after 5 days incubation. These results indicated that Fe^2+^ accelerated Hb auto-oxidation to the met-Hb form.

### 3.3 Lipid oxidation due to Hb, hemin, and iron

**Fig. 3:**
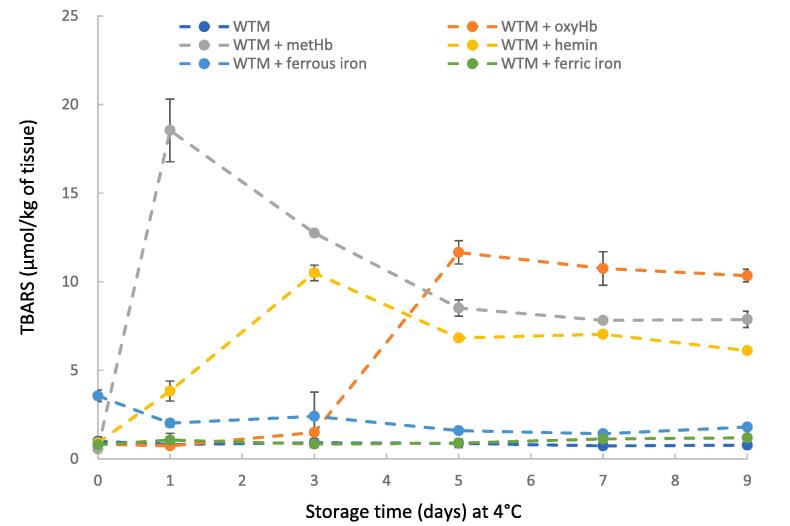
Thiobarbituric acid reactive substances (TBARS) values of washed turkey muscle (WTM) containing added oxyHb, metHb, hemin, ferrous iron, or ferric iron during 4 °C storage at pH 5.9. Each oxidant provided 40 µmol Fe per kg WTM (10 µmol Hb/kg WTM, 40 µmol hemin/kg WTM, 40 µmol ferrous chloride/kg WTM, and 40 µmol ferric chloride/kg WTM).

To compare the pro-oxidative activities of Hb-derivatives in lipid oxidation of the muscle system, oxyHb, metHb, hemin, ferrous iron, and ferric iron were separately added to WTM (Fig. 3). Among them, met-form Hb in WTM acted as the most potent pro-oxidant both in terms of onset and extent of lipid oxidation, showing maximum TBARS values at 24 h of storage. Addition of hemin as well as oxyHb to WTM also effectively incurred TBARS formation, with hemin causing TBARS formation more rapidly than oxyHb. In contrast, the pro-oxidative activities of ferrous and ferric iron were insufficient to initiate a chain reaction of lipid oxidation. However, at storage time 0, WTM with added ferrous iron had significantly higher TBARS values than oxyHb, metHb, hemin, and ferric iron.

### 3.4 Effects of NaCl and STPP on oxyHb-mediated lipid oxidation

**Fig. 4:**
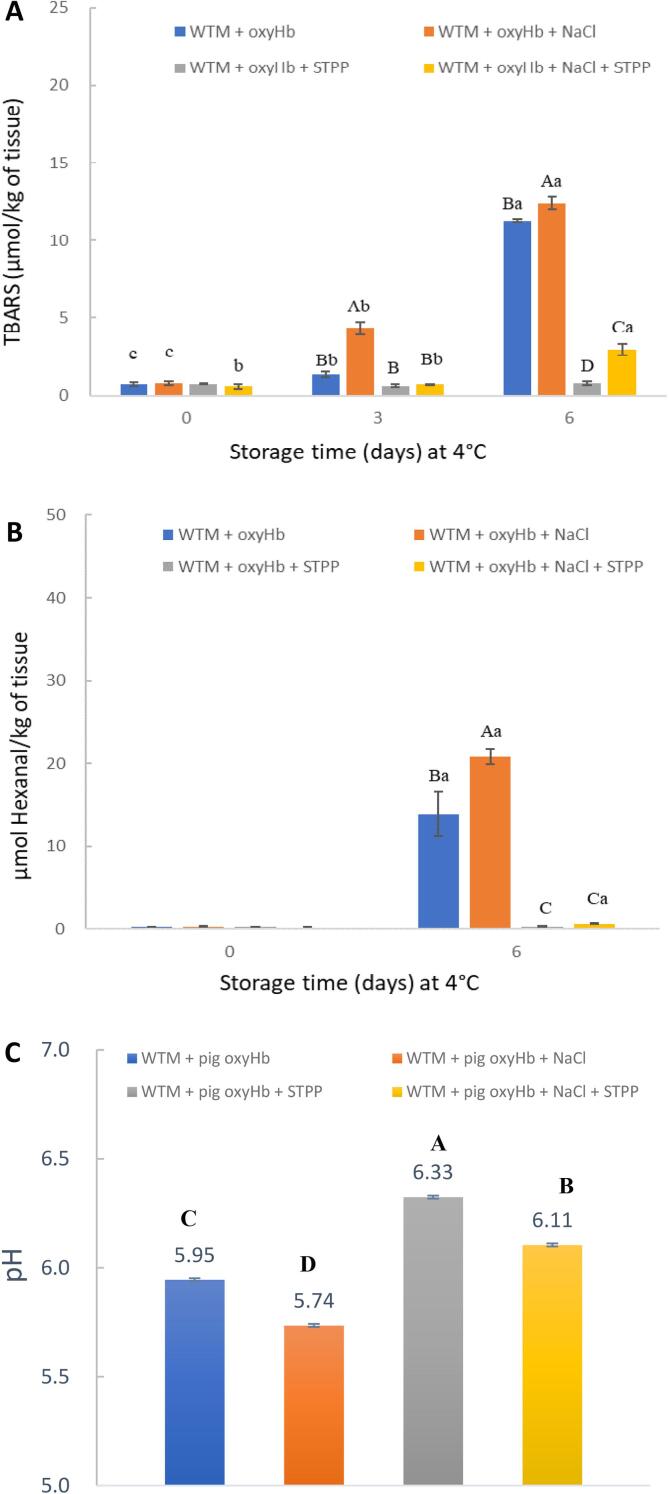
Effects of NaCl (1.5 %) and STPP (0.3 %) on TBARS (A), hexanal (B) and pH (C) of WTM with added oxyHb during storage at 4 °C. The pH of WTM was 5.9. OxyHb was added at 10 μmol/kg WTM. ^A-D^ Means with same letters within same storage time are not different (P > 0.05). ^a-c^ Means with same letters within same treatments are not different (P > 0.05).

Fig. 4 shows the effects of NaCl (1.5 %) and STPP (0.3 %) on oxyHb-mediated lipid oxidation of WTM during storage at 4 °C. TBARS values (Fig. 4A) and hexanal levels (Fig. 4B) of WTM with oxyHb significantly increased during storage. However, lipid oxidation of WTM with oxyHb was inhibited significantly by adding 0.3 % STPP. In contrast, NaCl significantly promoted oxyHb-mediated lipid oxidation in WTM. Furthermore, pro-oxidative activities of NaCl in WTM with oxyHb were predominantly inhibited by the addition of STPP.

### 3.5 Effect of ferrous iron on oxyHb-mediated lipid oxidation

**Fig. 5:**
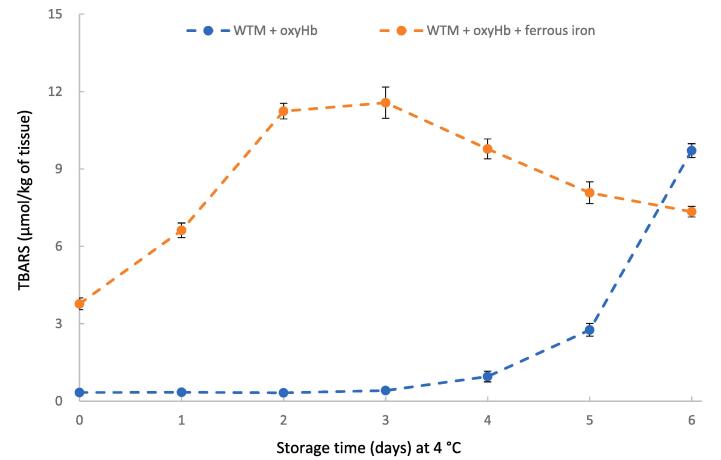
Effect of ferrous iron on oxyHb-mediated lipid oxidation of washed turkey muscle (WTM) during storage at 4 °C. Hb was added at 10 µmol/kg WTM. Ferrous iron concentration was 40 µmol/kg WTM and the pH was 5.9.

To identify the role of ferrous iron in oxyHb-mediated lipid oxidation, ferrous iron was added into WTM in the presence of oxyHb. As shown in Fig. 5, ferrous iron significantly promoted oxyHb-mediated lipid oxidation in WTM. Furthermore, the ferrous iron immediately caused the onset of TBARS formation.

## 4 Discussion

### 4.1 Lipid oxidation capacity of oxyHb, metHb, hemin, Fe^2+^ and Fe^3+^

The forms of iron play a critical role in their pro-oxidant ability in the muscle system (Wu, Richards, & Undeland, 2022). In our result (Fig. 3), Fe^3+^ did not show any pro-oxidant ability when using WTM. Similarly, Han et al. (1995) reported that FeCl_3_ did not promote lipid oxidation based on PV and TBARS in an aqueous beef residue model system. Additionally, Fe^2+^ also generated no detectable increase in TBARS during storage. This finding was in agreement with the data reported by Li et al. (2006), who found that adding FeCl_2_ did not initiate lipid oxidation in washed cod muscle system during the 8 days of storage on ice. The negligible pro-oxidant ability of both Fe^2+^ and Fe^3+^ in the present study may be attributed to the fact that the FeCl_2_ and FeCl_3_ were water-soluble and not accessible to lipid phases (e.g., phospholipid membranes). Specific types of chelators that form a complex with iron were necessary to facilitate iron-mediated lipid oxidation (Welch, Davis, & Aust, 2002). The washing process to prepare WTM has the potential to remove such a chelator. We also observed that the TBARS of WTM with Fe^2+^ was higher than other treatments at zero-time (Fig. 3). Manual mixing of Fe^2+^ into WTM may partition some of the Fe^2+^ into the lipid phase to degrade preformed lipid peroxides that facilitate lipid oxidation with formation of Fe^3+^ that is relatively inert as described by Huang et al. (1993) in sarcoplasmic reticulum.

The addition of free hemin in WTM immediately increased TBARS formation, and the observed lag time was<1 day (Fig. 3). This result agrees with previous data that hemin effectively stimulated TBARS formation in washed cod muscle, reaching a maximum after 2 days of ice storage (Grunwald & Richards, 2006). The effective pro-oxidant ability of hemin could be due to the intercalation of free hemin within phospholipid membranes by nonelectrostatic and electrostatic interactions (Giri et al., 2018). In doing so, hemin may readily decompose preformed lipid hydroperoxides and produce alkoxyl and peroxyl radicals that propagate lipid oxidation (Saito, Matsuoka, & Yamada, 2020). Depletion of hemopexin, which specifically inactivates hemin, was found to increase Hb-mediated lipid oxidation in human plasma (Yalamanoglu et al., 2018). Fig. 3 also shows that metHb had a more robust pro-oxidant ability than oxyHb in WTM. Similarly, our previous study found that the met form of Hb in turkey and pigs had a faster lipid oxidation rate than their oxy form in washed cod muscle (Wu, Yin, Zhang, & Richards, 2017). This finding could be attributed to the fact that metHb has more pronounced conformational dynamics that facilitate hemin release relative to oxyHb which retains its heme moiety (Bunn and Jandl, 1966, Sowole and Konermann, 2013). Furthermore, metHb promoted lipid oxidation more effectively than hemin (Fig. 3). These results could be due to the globin chains of metHb acting as hemin shuttles to deliver hemin into lipid phases, increasing the ability of hemin to oxidize lipids in WTM (Grunwald & Richards, 2006).

### 4.2 Effects of NaCl and STPP on oxyHb-mediated lipid oxidation

As an essential additive to meat, NaCl has been reported to promote lipid oxidation in raw and cooked meat at moderate concentrations (0.5–2.5 %) (Gheisari, Møller, Adamsen, & Skibsted, 2010). However, the effect of NaCl on the pro-oxidant activities of heme proteins is unclear. From our findings (Fig. 4), both TBARS and hexanal results indicated that the addition of NaCl (1.5 %) increased oxyHb-mediated lipid oxidation in WTM over 6 days of storage. The possible mechanisms of the pro-oxidant action of NaCl at 1.5 % in the present study may involve diverse pathways. Firstly, NaCl increased the oxidation of oxyHb to form metHb in Fig. 1B, and the metHb showed a more effective promoting ability than oxyHb (Fig. 3). Secondly, the pro-oxidant effect of NaCl can also be partly attributed to the decreased pH effect of added NaCl, dropping pH by approximately 0.2 units from 5.95 to 5.75 in the washed muscle (Fig. 4C). Bovine Hb mediated lipid oxidation was increased 60-fold as pH was decreased from 6.7 to 6.3, indicating relatively modest decreases in pH can significantly increase lipid oxidation due to Hb (Yin, Zhang, & Richards, 2017).

The addition of STPP strongly decreased oxyHb-mediated lipid oxidation over 6 days of storage, as shown in Fig. 4. One reason could be the increase in pH of WTM in the presence of STPP. Fig. 4C shows that the pH increased from 5.95 to 6.33 when 0.3 % STPP was added to WTM. This pH increase substantially decreased the formation of metHb from oxyHb when storing Hb in solution (Fig. 1A). The slower formation of metHb at higher pH will delay the onset of lipid oxidation, noting the improved ability of metHb to promote lipid oxidation than oxyHb (Fig. 3).

Another reason STPP strongly inhibited oxyHb-mediated lipid oxidation in WTM could involve the ability of STPP to chelate Fe^2+^ and thereby inhibit the ability of Fe^2+^ to incur oxidative reactions. It was previously shown that Fe^2+^ increased the formation of oxidized heme proteins and lipid oxidation in a pork homogenate system (Zhou, Jongberg, Zhao, Sun, & Skibsted, 2016). Our findings are consistent with a pro-oxidant effect of Fe^2+^ in the presence of heme proteins; in that, addition of Fe^2+^ to oxyHb increased Hb auto-oxidation (Fig. 2), and added Fe^2+^ increased the ability of oxyHb to promote lipid oxidation in WTM (Fig. 5). Thus, part of the antioxidant mechanism of STPP may involve chelating Fe^2+^ that would otherwise convert oxyHb to metHb.

One final reason STPP strongly inhibited oxyHb-mediated lipid oxidation in WTM could be that STPP alters the physical structure of muscle by dissociating actin from myosin (Speroni, Szerman, & Vaudagna, 2014). The ability of STPP to change the physical location of hemin that becomes released from metHb in the washed muscle should be considered a possible antioxidative mechanism. Previously, the lag phase prior to the exponential increase in lipid oxidation reciprocally shortened as more hemin was bound to the insoluble matrix of washed muscle (Tatiyaborworntham, Yin, & Richards, 2021). Future work should examine the ability of STPP to affect the association of metHb and hemin with the insoluble fraction of the washed muscle.

Our results (Fig. 4) also show that STPP can weaken the pro-oxidative effect of NaCl on Hb-mediated lipid oxidation in WTM. This could be attributed to the higher pH in the WTM and lower auto-oxidation rate of oxyHb in the presence of STPP. Table 1 shows that the pH value of NaCl + STPP was 6.1, but the only NaCl treatment was pH 5.7. In addition, the auto-oxidation rate of Hb with NaCl + STPP was significantly lower than only NaCl (0.93 vs 1.18), as can be seen in Supplementary material Table S2.

## 5 Conclusions

Among Hb derivatives, metHb in WTM acted as the most effective pro-oxidant, followed by hemin and oxyHb. However, free iron (Fe^2+^ and Fe^3+^) did not show pro-oxidant activity in WTM. Additionally, the addition of NaCl significantly increased the oxyHb-mediated lipid oxidation. The decrease in pH value and increase of % metHb partly explained why NaCl increased the pro-oxidant activity of oxyHb. STPP inhibited oxyHb-mediated lipid oxidation. The increase of pH value, inactivation of iron, and inhibition of the formation of metHb seem to explain the inhibiting effect of STPP on oxyHb-mediated lipid oxidation in WTM. These studies provide foundational knowledge for understanding the effect of NaCl and STPP on lipid oxidation in meat. Furthermore, these findings could provide foundational information for controlling lipid oxidation in processing meat products involving NaCl and STPP.

## Declaration of Competing Interest

The authors declare that they have no known competing financial interests or personal relationships that could have appeared to influence the work reported in this paper.

## Data Availability

Data will be made available on request.
