# Supplementary material for: Effects of sodium chloride and sodium tripolyphosphate on the prooxidant properties of hemoglobin in washed turkey muscle system
Source: Food Chem X. 2022 Oct 13;16:100480. doi: 10.1016/j.fochx.2022.100480 (PMC9583034; doi:10.1016/j.fochx.2022.100480)
Supplement: Supplementary data 1 [file mmc1.docx]

**Supplementary data**

**Table S1.** Relative rates of auto-oxidation of pig Hb during incubation at 37°C, as affected by pH

|  | **pH** | | | | |
| --- | --- | --- | --- | --- | --- |
|  | 5.0 | 5.4 | 5.8 | 6.2 | 6.6 |
| Mean | 3.97^a^ | 2.19^b^ | 1.00^c^ | 0.40^d^ | 0.16^e^ |
| STD | 0.03 | 0.03 | 0.04 | 0.01 | 0.01 |

Relative rates of heme protein auto-oxidation were based on the slopes (Figure 1A), which were made of data of percent met heme protein from 0 to 60% with time. ^a-e^ Means with same superscripts within same row are not different (p > 0.05).

**Table S2.** Relative rates of auto-oxidation of pig Hb during incubation at 37^o^C, as affected by sodium chloride (NaCl) and sodium tripolyphosphate (STPP)

|  | CTL | T1 | T2 | T3 | T4 | T5 | T6 | T7 |
| --- | --- | --- | --- | --- | --- | --- | --- | --- |
| NaCl (%) |  | 1.0 | 1.5 | 2.0 |  |  |  | 1.5 |
| STPP (%) |  |  |  |  | 0.1 | 0.3 | 0.5 | 0.3 |
| Mean | 1.00^c^ | 1.13^b^ | 1.18^ab^ | 1.20^a^ | 1.00^c^ | 0.91^d^ | 0.92^d^ | 0.93^d^ |
| STD | 0.01 | 0.04 | 0.03 | 0.03 | 0.05 | 0.03 | 0.02 | 0.01 |

Relative rates of heme protein auto-oxidation were based on the slopes (Figure 1B and 1C), which were made of data of percent met heme protein from 0 to 60% with time. ^a-d^ Means with same superscripts within same row are not different (p > 0.05).
